# Management of Advanced Peri-Implantitis with Staged Explantation and Delayed Re-Implantation in the Esthetic Zone

**DOI:** 10.3390/dj14040212

**Published:** 2026-04-07

**Authors:** Alexandru Spînu, Felicia Manole, Alexandru Burcea, Cristina-Crenguţa Albu, Adrian Argint, Lavinia-Florica Mărcuț, Roxana Daniela Brata, Alexia Manole, Claudia Florina Bogdan-Andreescu

**Affiliations:** 1 Doctoral School, Faculty of Medicine and Pharmacy, University of Oradea, 410068 Oradea, Romania; spinu.alexandru@didactic.uoradea.ro; 2 Spinu Dental Clinic, Spinu Learning, 410155 Oradea, Romania; adrianstomatolog@gmail.com; 3 Surgical Disciplines Department, Faculty of Medicine and Pharmacy, University of Oradea, 410068 Oradea, Romania; fmanole@uoradea.ro (F.M.); lmarcut@uoradea.ro (L.-F.M.); 4 Department of Speciality Disciplines, “Titu Maiorescu” University, 031593 Bucharest, Romania; claudia.andreescu@prof.utm.ro; 5 Department of Genetics, Faculty of Dentistry, “Carol Davila” University of Medicine and Pharmacy, 020021 Bucharest, Romania; 6 Medical Disciplines Department, Faculty of Medicine and Pharmacy, University of Oradea, 410068 Oradea, Romania; brata.roxanadaniela@didactic.uoradea.ro; 7 Faculty of Medicine and Pharmacy, University of Oradea, 410068 Oradea, Romania; manole.alexia@student.uoradea.ro

**Keywords:** biologically driven treatment planning, connective tissue graft, enamel matrix derivative, esthetic zone reconstruction, implant explantation, interproximal bone peak, non-submerged implant placement, peri-implantitis management, periodontal regeneration, staged implant therapy

## Abstract

**Background/Objective:** Advanced peri-implantitis presents a significant challenge in contemporary implant dentistry and sometimes necessitates implant removal when regenerative therapies are no longer reliable. Protocols for staged bone reconstruction, re-implantation, and definitive prosthetic rehabilitation following peri-implantitis continue to evolve. This study aims to present a clinical case of advanced peri-implantitis with vertical interproximal bone loss managed with a staged surgical and prosthetic approach and review current concepts in implant removal, bone regeneration, re-implantation, and soft-tissue management. **Methods:** A patient with peri-implantitis affecting two maxillary implants underwent treatment over one year. The initial surgical stage included removal of the failing implants and reconstruction of the defects using guided bone regeneration with a composite graft of 50% xenogeneic bone substitute and 50% autogenous bone, covered by a barrier membrane. After six months of healing, a second surgical stage was performed, involving placement of two new implants in positions 2.2 and 2.4, additional bone augmentation, and soft tissue grafting to enhance soft tissue volume and the width of keratinized gingiva following mucogingival line rebounce. After an additional six months of osseointegration, full maxillary prosthetic rehabilitation was completed in August 2025. **Results:** Clinical and radiographic assessments demonstrated successful bone regeneration, stable implant integration, adequate peri-implant soft-tissue conditions, and favorable functional and esthetic outcomes at follow-up. The case is discussed in the context of current evidence regarding indications for implant removal, regenerative strategies after explantation, timing of re-implantation, and the importance of keratinized gingiva and prosthetic design in long-term peri-implant health. **Conclusions:** Staged explantation, guided bone regeneration, delayed re-implantation, and comprehensive soft-tissue and prosthetic management may represent a viable treatment strategy in selected cases of advanced peri-implantitis.

## 1. Introduction

Dental implant therapy is a widely adopted and predictable method for replacing missing teeth; however, failures and complications remain frequent [1]. The long-term success of implant-supported prostheses is strongly linked to the absence of inflammation in peri-implant soft tissues [2]. As the use of dental implants increases, the prevalence of peri-implant diseases has also risen, with peri-implantitis rates reported between 28% and 51% in various studies [3]. Systematic reviews and meta-analyses have found peri-implantitis prevalence rates of 19.53% at the patient level and 12.53% at the implant level in 2022 [4], and 25.0% at the patient level and 18.0% at the implant level in 2025 [5]. The latter study also reported a high prevalence of peri-implant mucositis, affecting 63.0% of patients and 59.2% of implants [5].

The rising prevalence of peri-implant diseases presents a significant challenge in modern implant dentistry, as inflammation of peri-implant tissues is linked to progressive bone loss and potential implant failure [6].

Despite extensive research, peri-implantitis management remains complex and controversial. Various non-surgical and surgical modalities have been proposed, such as mechanical debridement, chemical and laser-based decontamination, and regenerative surgical techniques [7,8,9]. While these therapies may be effective in early or moderate disease stages, current evidence suggests that outcomes in advanced peri-implantitis are highly variable and often unpredictable, especially in cases with extensive circumferential bone loss, implant surface contamination, or unfavorable implant positioning [10,11,12,13].

In advanced peri-implantitis cases with more than 50% bone loss, clinicians must decide between attempting to save or remove the affected implant. Systematic reviews and consensus reports indicate that, when prognosis is poor, implant removal followed by site reconstruction yields more predictable long-term outcomes than repeated regenerative attempts with uncertain efficacy [11,14]. As a result, there is increasing interest in staged bone regeneration, delayed re-implantation, and subsequent prosthetic rehabilitation after peri-implantitis [15,16,17].

Guided bone regeneration (GBR) represents the primary approach for reconstructing bone defects following implant removal, with composite grafting strategies combining autogenous and xenogeneic materials demonstrating favorable biological and volumetric outcomes [18,19,20]. Contemporary treatment protocols emphasize prosthetically driven implant placement, optimization of keratinized mucosa, and structured maintenance programs to minimize disease recurrence [21,22,23,24,25,26].

Comprehensive clinical reports detailing staged protocols that integrate implant removal, bone regeneration, delayed re-implantation, and definitive prosthetic rehabilitation for advanced peri-implantitis are currently limited. Extensive bone loss and unfavorable implant positioning can compromise the predictability of regenerative peri-implantitis therapy. In such cases, implant removal followed by staged reconstruction may offer a more biologically predictable solution. This report presents the staged management of advanced peri-implantitis in the esthetic zone. It encompasses implant explantation, guided bone regeneration, delayed re-implantation, and prosthetic rehabilitation. The report examines the biological and clinical considerations relevant to this treatment strategy.

## 2. Case Presentation

### 2.1. Patient History and Diagnosis

**Figure 1:**
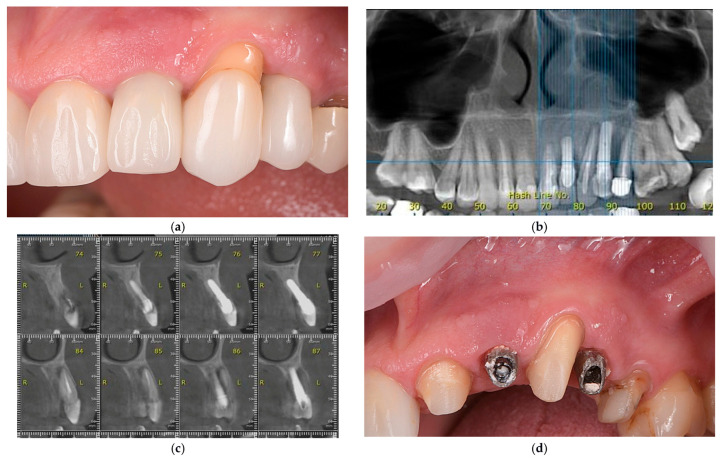
Representative clinical and radiographic images at presentation. (**a**) Clinical aspect of the implant-supported crowns and adjacent crowns on natural teeth; (**b**,**c**) CBCT scan confirming bone loss around implants (The letters “R” and “L” indicate the right and left sides, respectively); (**d**) Aspect of implant abutments and surrounding soft tissues after crown removal.

A 50-year-old female patient presented to Spînu Dental Clinic with concerns regarding an unsatisfactory prosthetic restoration in the left maxilla (Figure 1a). Medical history indicated that two post-extraction dental implants were placed in 2018 and immediately loaded with cement-retained crowns. The patient expressed dissatisfaction with the aesthetic outcome, specifically the appearance of the crowns in relation to adjacent natural teeth and peri-implant soft tissues. Additionally, recurrent purulent secretion from the soft tissue was reported in the left maxillary region.

The patient was in good general health, with no relevant systemic diseases or medical conditions. She was not taking any long-term medications, reported no known allergies, and was a non-smoker. Dental history included caries experience and fixed prosthodontic treatments, with no history of periodontitis.

Intraoral examination identified implant-supported crowns in the left maxillary region, replacing the lateral incisor and first premolar, with significant esthetic deficiencies. Discrepancies in color, translucency, and morphology were noted between the implant-supported crowns and adjacent natural teeth. The peri-implant soft tissues exhibited gingival discoloration and irregular architecture, lacking harmonious gingival scalloping and demonstrating deficient papilla formation compared to neighboring teeth. Periodontal probing revealed increased depths exceeding 8 mm, with a maximum of 15 mm on the distal aspect of the canine and 12 mm on the mesial aspect of the second premolar.

Based on the 2017 World Workshop criteria, the clinical and radiographic findings were consistent with advanced peri-implantitis, characterized by probing depths ≥8 mm, suppuration, and radiographic bone loss exceeding 50% of the implant length [27].

### 2.2. Imaging and Treatment Planning

Radiographic evaluation using cone-beam computed tomography (CBCT) was conducted to further assess peri-implant hard-tissue conditions. CBCT analysis confirmed a combined vertical and horizontal alveolar ridge defect, with a pronounced deficiency of the buccal cortical plate at both implant sites (Figure 1b,c). These findings aligned with a Class III peri-implant dehiscence-type defect, characterized by both infraosseous and horizontal components [28]. The significantly reduced peri-implant bone volume provided inadequate support for the overlying soft tissues, which corresponded with the unfavorable clinical presentation, including increased probing depths, soft-tissue instability, and esthetic deficiencies.

CBCT evaluation also identified significant bone loss affecting the adjacent natural teeth, specifically the canine and second premolar. Radiographic analysis revealed vertical and horizontal alveolar bone resorption along the approximal and buccal aspects of these teeth, resulting in significant exposure of the radicular surfaces. The pattern and extent of bone loss indicated secondary involvement due to the peri-implant inflammatory process and inadequate prosthetic contours, rather than primary periodontal disease. These findings compromised the soft-tissue architecture and contributed to the observed esthetic disharmony, supporting the necessity for a comprehensive hard- and soft-tissue reconstructive approach.

Based on the clinical and radiological findings, multiple treatment options were presented to the patient. Nonsurgical peri-implant therapy with prosthetic modification was associated with a limited prognosis, as it would address inflammation but not the underlying bone defect. Surgical peri-implant reconstructive therapy, including guided bone regeneration (GBR) and soft-tissue augmentation, offered a moderate prognosis for tissue stability and esthetics; however, complete vertical bone reconstruction in the esthetic zone remained unpredictable. Implant removal followed by ridge augmentation and delayed reimplantation provided the most favorable long-term prognosis, enabling comprehensive correction of hard- and soft-tissue deficiencies and improved implant placement, albeit with increased treatment duration, cost, and surgical morbidity.

The patient selected a surgical approach that included removal of the existing implants, hard- and soft-tissue augmentation, and subsequent prosthetic rehabilitation of the maxilla. This choice was primarily motivated by concerns regarding esthetics and the desire for a predictable, long-term outcome. Although extraction of the adjacent natural teeth could have been considered, given the extent of the bone defects, the patient strongly preferred preservation of the canine and premolar. Consequently, following a comprehensive clinical and radiological evaluation, a tooth-preserving treatment strategy was implemented.

Prior to treatment initiation, the patient was thoroughly informed about the proposed procedures, including potential risks, benefits, alternative options, and possible complications. Written informed consent was obtained for all clinical and surgical interventions, as well as for the use of anonymized clinical data and images for scientific publication, in accordance with the Declaration of Helsinki.

After removal of the implant-supported crowns, clinical inspection revealed thin and irregular peri-implant soft tissues, loss of keratinized mucosa at both implant sites, and residual cement deposits (Figure 1d).

### 2.3. Stage One Surgery: Implant Removal and Guided Bone Regeneration

The first surgical stage was done in August 2024 and involved implant explantation, root planing of the adjacent teeth, and decontamination of the radicular surfaces. This was followed by guided bone regeneration using autologous bone harvested from the maxillary tuberosity, mixed in a 50:50 ratio with a collagenated porcine-derived xenograft material, and covered with a resorbable barrier membrane.

#### 2.3.1. Preoperative Preparation

The patient received antibiotic prophylaxis with amoxicillin and clavulanic acid 875/125 mg (Augmentin^®^, GlaxoSmithKline, London, UK), prescribed at a dosage of one tablet every 12 h, starting 24 h before surgery and continued for seven days.

Immediately before anesthesia, the patient performed an oral rinse with a 0.2% chlorhexidine solution to reduce the bacterial load in the oral cavity [29]. Local anesthesia was achieved using articaine hydrochloride 4% with epinephrine 1:100,000 (Ubistesin Forte^®^, 3M ESPE, Seefeld, Germany), administered via posterior superior alveolar and greater palatine nerve blocks.

#### 2.3.2. Surgical Steps

**Figure 2:**
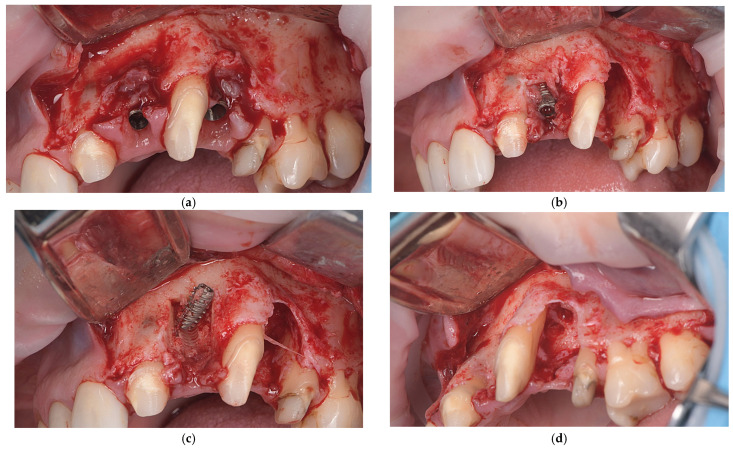

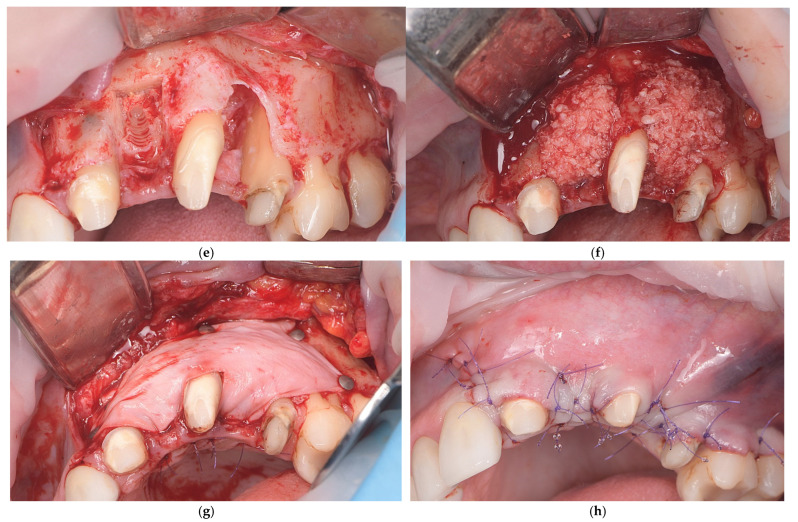
Representative images of stage one surgery. (**a**) Intraoperative view after flap elevation demonstrating the presence of peri-implant granulation tissue; (**b**) Explantation of one implant, demonstrating the residual bone defect, and the mesial aspect of the root surface of tooth 2.5 exposed within the defect area; (**c**) Explantation of second implant and remaining bone defect after explantation; (**d**) Intraoperative view showing the distal aspects of the exposed root surfaces of teeth 2.1. and 2.3.; (**e**) Bone defects and exposed roots of adjacent teeth after EDTA gel treatment; (**f**) Augmentation of the bone defects using a particulate bone graft following implant explantation; (**g**) Stabilization of bone graft with a resorbable barrier membrane with fixation; (**h**) Flap closure and suturing.

A crestal incision with vertical releasing incisions was made to access the implant sites (2.2. and 2.4.), adjacent teeth (2.1., 2.3., and 2.5.), and left maxillary tuberosity for impacted third molar extraction and bone harvesting. A full-thickness mucoperiosteal flap was then elevated to expose the left maxilla (Figure 2a).

After flap elevation, granulation tissue was observed around the implants, and the adjacent teeth showed exposed radicular surfaces, consistent with preoperative radiological findings. The granulation tissue was carefully removed. The implant at position 2.4. was explanted using a reverse-torque device (MIS Implants Technologies Ltd., Misgav, Israel), while the implant at 2.2. was removed with a trephine bur followed by reverse-torque explantation (Figure 2b,c). The exposed root surfaces of the adjacent teeth within the bone defects were decontaminated with doxycycline paste for two minutes, planed with Gracey curettes, and conditioned with 24% EDTA gel (PrefGel^®^, Straumann AG, Basel, Switzerland) (Figure 2d,e). Enamel matrix derivative (Emdogain^®^, Straumann AG, Basel, Switzerland) was applied to the exposed root surfaces of teeth 2.1, 2.3, and 2.5, then covered with autogenous bone chips from the left maxillary tuberosity to promote periodontal regeneration.

The bone defects were filled with a particulate graft composed of a 50:50 mixture of autologous bone from the maxillary tuberosity and small-particle (0.25–1.00 mm) collagenated porcine-derived xenograft material (THE Graft™, Purgo Biologics Inc., Seongnam-si, Gyeonggi-do, Republic of Korea) (Figure 2f). The exposed root surfaces of teeth 2.1., 2.3., and 2.5. were covered exclusively with autologous bone chips to promote periodontal regeneration, due to their osteogenic and osteoinductive properties and favorable interaction with the periodontal ligament and root surface [30].

After graft placement, the augmented area was covered with a resorbable collagen membrane (THE Cover™, Purgo Biologics Inc., Seongnam-si, Gyeonggi-do, Republic of Korea) and stabilized with fixation pins to maintain space and immobilize the graft (Figure 2g). On the palatal aspect, the membrane was secured with 6-0 polyglycolide-caprolactone (PGCL) monofilament resorbable sutures (Monofast^®^, Assut Europe S.p.A., Pescara, Italy). The membrane fully covered the grafted region and extended beyond the defect margins. The flap was mobilized to achieve tension-free primary closure, then repositioned and sutured with 4-0 PTFE sutures (Biotex™, Purgo Biologics Inc., Seongnam-si, Gyeonggi-do, Republic of Korea) (Figure 2h).

#### 2.3.3. Postoperative Care

Postoperative management included administration of nimesulide 100 mg (Aulin^®^, Angelini Pharma, București, Romania) every 12 h to control pain and inflammation. The patient was advised to apply cold packs during the first 48 h and to maintain a soft diet until suture removal. Oral hygiene was maintained with saline rinses while avoiding toothbrushing at the surgical site. Strenuous physical activity was restricted for 2–4 weeks to protect graft stability. Mild discomfort and swelling were considered normal postoperative findings.

### 2.4. Healing Phase

The healing period following bone augmentation was uneventful, with no signs of infection or membrane exposure. Sutures were removed 12 days after surgery. During the six-month healing phase, the patient was rehabilitated with a tooth-supported provisional fixed restoration, delivered two days after surgery, and designed to avoid any pressure on the grafted site. The provisional bridge was fabricated with pontics maintained in no contact with the augmented ridge, and the occlusion was carefully adjusted to eliminate functional loading.

### 2.5. Stage Two Surgery: Re-Implantation and Soft-Tissue Management

**Figure 3:**
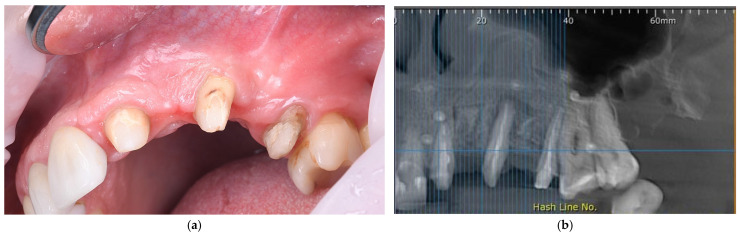

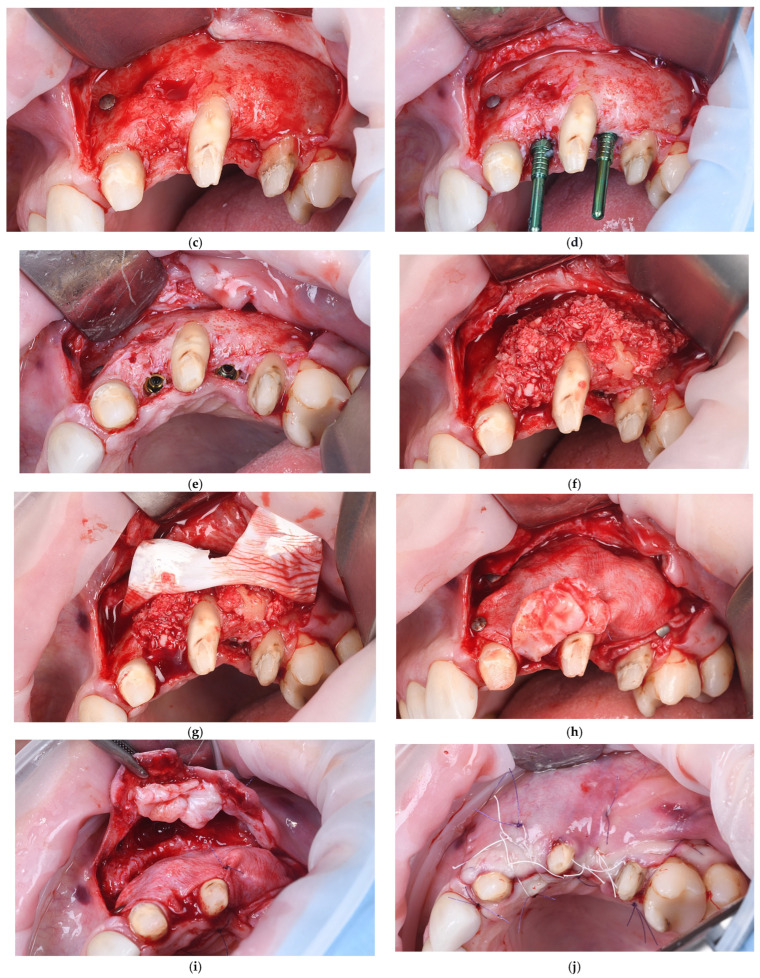
Representative images of stage two surgery. (**a**) Clinical view of the healed alveolar ridge six months after GBR, showing ridge volume prior to implants placement, with corticalized bone gain; (**b**) CBCT assessment six months after GBR demonstrating bone regeneration; (**c**) Intraoperative view after membrane removal illustrating newly regenerated bone and favorable ridge morphology; (**d**) Intraoperative view illustrating osteotomy preparation and assessment of implant parallelism; (**e**) Placement of two hybrid-level dental implants; (**f**) Peri-implant bone augmentation using particulate graft material; (**g**) Stabilization with a barrier membrane; (**h**) Connective tissue graft positioned to improve peri-implant soft-tissue volume and quality; (**i**) Fixation of a connective tissue graft; (**j**) Flap closure and suturing.

Six months after the initial surgery, a CBCT scan was performed to assess the alveolar ridge. Clinical and CBCT evaluations confirmed sufficient new bone formation for standard-diameter implant placement (Figure 3a,b). The ridge width measured approximately 8 mm, with CBCT showing nearly complete bone coverage of the distal root surface of the canine, the mesial surface of the second premolar, and the distal surface of the central incisor. Periodontal probing depths were reduced to 4 mm at the canine and second premolar sites, indicating favorable healing.

Preoperative preparation matched the first surgical stage. A trapezoidal mucoperiosteal incision exposed the grafted area and adjacent teeth. Flap elevation revealed mature cortical bone at the augmented site (Figure 3c). Osteotomies for standard-length, standard-diameter implants were prepared (Figure 3d), and two bone-level Prama^®^ implants (Sweden & Martina, Due Carrare, Italy) were placed using a non-submerged, one-stage approach (Figure 3e). The implants had anodized transmucosal necks measuring 2.8 mm at site 2.2. and 3.8 mm at site 2.4.

Additional bone augmentation with slight supracorrection was performed to optimize ridge contours, using a collagenated porcine-derived xenograft mixed 1:1 with autologous bone from the external oblique ridge (Figure 3f). As before, the roots of adjacent teeth were covered exclusively with autogenous bone. The grafted area was then covered with a resorbable collagen barrier membrane (Figure 3g). A closed connective tissue graft from the palate was also performed to enhance soft-tissue volume and increase keratinized gingival width after mucogingival rebound around the implant sites (Figure 3h,i). The flap was stabilized with horizontal mattress and simple interrupted sutures for tension-free primary closure, using 4-0 PTFE sutures (Figure 3j).

**Figure 4:**
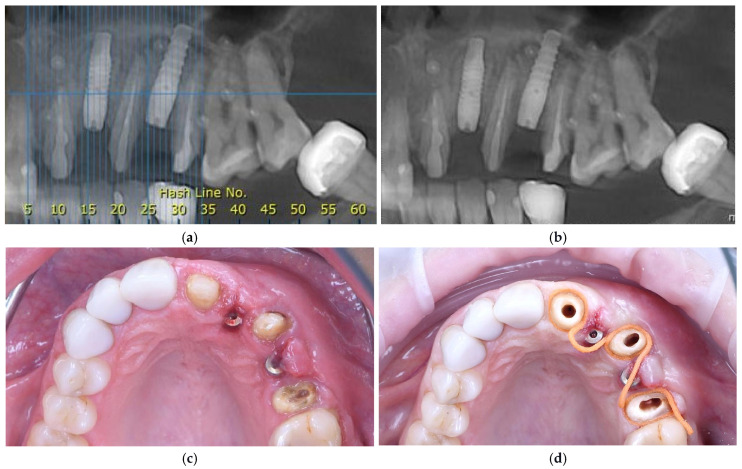
Representative clinical and radiographic images at presentation. (**a**) CBCT scan obtained after the second surgical intervention, demonstrating stable implant positioning and adequate horizontal bone augmentation; (**b**) CBCT scan prior to implant uncovering, confirming a stable and uniform width of the alveolar ridge; (**c**) Occlusal view of the maxilla after implant uncovering, illustrating the soft tissue contours prior to prosthetic rehabilitation; (**d**) Root surface preparation of adjacent teeth for post-and-core restorations.

A postoperative CBCT confirmed an alveolar ridge width of approximately 8 mm at the augmented sites, indicating successful horizontal bone augmentation (Figure 4a). Postoperative care and the healing phase were identical to those of the first surgical stage.

### 2.6. Stage Three Surgery: Implant Exposure and Soft-Tissue Management

Six months after the second surgical intervention, a CBCT examination was performed to evaluate the alveolar ridge. The CBCT analysis demonstrated adequate buccal bone volume to support the peri-implant soft tissues, allowing for a functional and esthetic prosthetic restoration (Figure 4b). Owing to the connective tissue graft, mucogingival rebound was avoided, and the vestibular depth was preserved without deformation. Depths on probing were 3 mm for 2.3. and 2.5.

The outcomes of the regenerative procedures were evaluated through clinical and radiographic parameters, including soft-tissue healing, gingival margin stability, absence of inflammation, and radiographic evidence of bone fill. Periodontal probing depth (PPD) was also recorded on the adjacent natural teeth during follow-up to assess periodontal stability.

Periodontal probing depth at the teeth adjacent to the implant sites was monitored. The maxillary canine showed a reduction from 12 mm at baseline to 4 mm and 3 mm at follow-up, indicating a favorable response to the regenerative therapy.

At this stage of therapy, the implants were surgically uncovered, healing caps were placed, and an impression for a temporary restoration was taken.

### 2.7. Prosthetic Rehabilitation and Follow-Up

At this stage, a gingivectomy combined with a limited flapless osteotomy at the implant level was performed using a piezoelectric device (CVDentus, São José dos Campos, Brazil) to re-establish the peri-implant biological width and to create adequate vertical space for the definitive prosthetic crowns.

**Figure 5:**
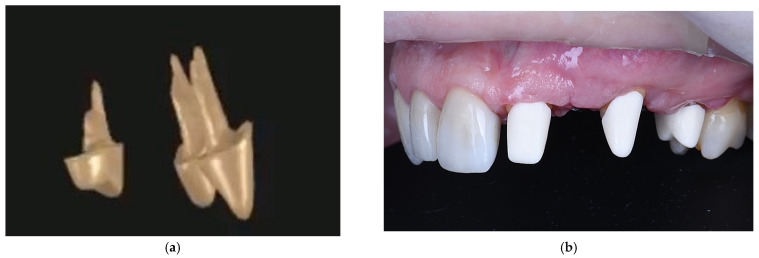

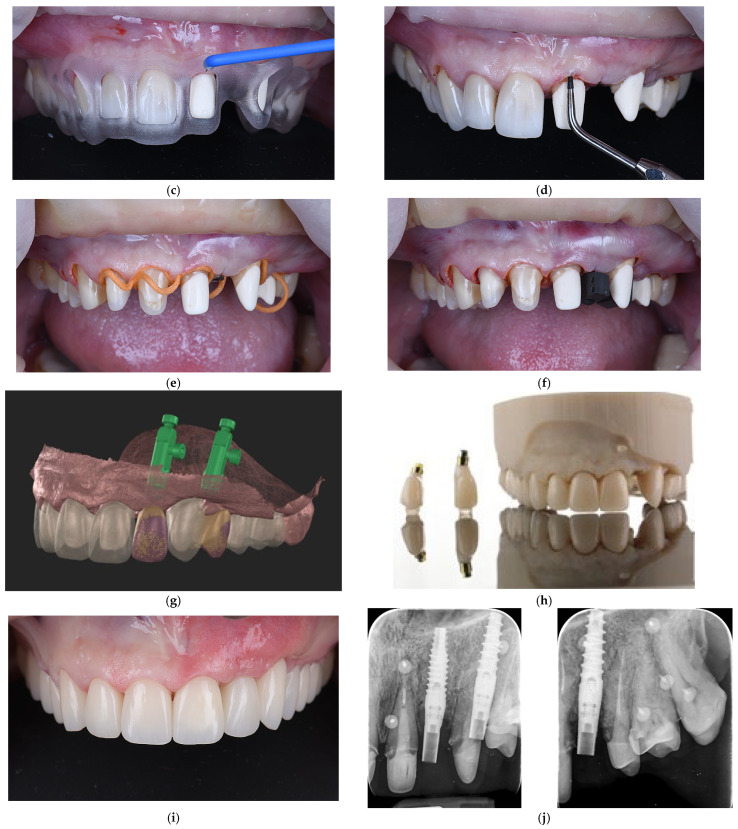

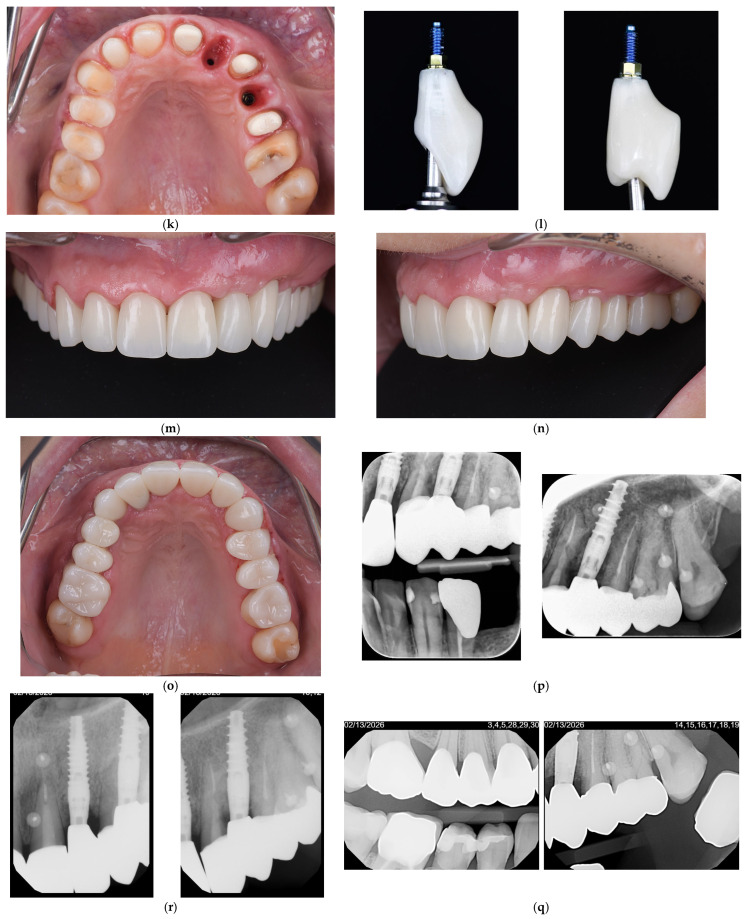
Representative clinical and radiographic images at presentation. (**a**) Three-dimensional aspect of the post-and-core restorations for teeth 2.1., 2.3., and 2.5.; (**b**) Post-and-core restorations cemented and buccal aspect of the vestibule after GBR, prior to the restorative stage; (**c**) Use of a transparent gingivectomy guide to define the gingival margin level and achieve soft tissue symmetry; (**d**) Final refinement of the tooth surfaces with ultrasonic instruments; (**e**) Buccal clinical view showing placement of a gingival retraction cord for soft tissue displacement; (**f**) Intraoral view before impression making, showing gingival retraction and transfer abutments positioned; (**g**) Virtual planning view of temporary crowns showing implant positions with scan bodies and the surrounding soft tissue anatomy; (**h**) Temporary prosthetic restorations illustrating the esthetic and functional outcome; (**i**) Frontal clinical view of the definitive restorations; (**j**) Periapical radiographic assessment of post-and-core restorations and prosthetic abutment; (**k**) Occlusal view demonstrating peri-implant soft tissue maturation and keratinized gingiva around the implant sites before final restorations; (**l**) Extraoral view of the final screw-retained implant-supported crowns; (**m**) Frontal clinical view of the final implant-supported and tooth-supported restorations; (**n**) Lateral clinical view of the final prosthetic rehabilitation, showing stable peri-implant soft tissues and harmonious gingival architecture; (**o**) Occlusal clinical view of the maxillary arch after final prosthetic rehabilitation; (**p**) Periapical radiographs showing the final implant-supported restoration and adjacent tooth-supported restorations; (**r**) Periapical radiograph obtained at six-month follow-up, demonstrating stable peri-implant bone levels; (**q**) Bitewing radiograph obtained at six-month follow-up.

For esthetic and functional reasons, a full maxillary arch rehabilitation with individual zirconia crowns was planned. Prosthetic treatment began with the reconstruction of the teeth adjacent to the implants (2.1., 2.3., and 2.5.) using zirconia post-and-core restorations (Figure 5a,b). A transparent prosthetic guide was employed to determine the appropriate level of the free gingival margin and to achieve symmetry with the contralateral dentition (Figure 5c). Final refinement of the tooth preparations was performed accordingly using ultrasonic instruments (CVDentus, São José dos Campos, Brazil) for precision finishing (Figure 5d).

Gingival retraction was achieved using retraction cords to temporarily displace the marginal gingiva and allow adequate exposure of the finishing lines prior to impression taking (Figure 5e,f). Provisional restorations were digitally designed (Figure 5g) and milled from polymethyl methacrylate (PMMA) (Figure 5h,i). Special attention was given to the design of the provisional restorations to establish a new emergence profile that supports the gingival tissues around both teeth and implants, in accordance with the biologically oriented preparation technique (BOPT) protocol. Periapical radiographs confirmed proper adaptation of the provisional restorations and prosthetic components (Figure 5j).

Three weeks after delivery of the provisional restorations, clinical evaluation revealed satisfactory maturation of the peri-implant soft tissues and an adequate width of keratinized gingiva (3 mm around the implant neck), allowing progression to the definitive prosthetic phase (Figure 5k). The final zirconia restorations were subsequently delivered, demonstrating favorable esthetic integration and functional outcomes (Figure 5l–n). The implant-supported restorations were fabricated as screw-retained zirconia crowns on LMDT (Low-MDT—Management of Deep Tissue) abutments (Sweden & Martina, Due Carrare, Italy), with an emergence profile transitioning from a concave subgingival contour to a slightly convex transmucosal profile. The radiographic assessment confirmed proper adaptation of the definitive restorations and stable peri-implant bone conditions (Figure 5o).

The patient maintained excellent oral hygiene, with a plaque index (PI) of 0, and no bleeding on probing (BOP) was observed after resolution of the initial inflammatory condition. At the six-month follow-up, radiographic evaluations demonstrated stable peri-implant and periodontal conditions. The periapical and bitewing radiographs confirmed the maintenance of marginal bone levels around the re-implanted fixtures, with no signs of peri-implant radiolucency, progressive bone loss, or inflammatory changes (Figure 5q,r). The adjacent natural teeth exhibited stable periodontal support and absence of periapical pathology. The prosthetic components showed proper adaptation and occlusal stability, indicating successful functional and biological integration at this stage of follow-up.

This case report was conducted and reported in accordance with the CARE (CAseREport) guidelines, with compliance summarized in Table 1.

## 3. Discussion

Managing advanced peri-implantitis is among the most complex challenges in contemporary implant dentistry. Although various non-surgical and surgical interventions have been described, current evidence demonstrates that regenerative peri-implantitis therapy is increasingly unpredictable in cases with circumferential bone loss, implant surface contamination, and suboptimal implant positioning. In these scenarios, implant removal followed by staged reconstruction may offer a more biologically sound and predictable approach.

Regenerative peri-implantitis therapy without implant removal can yield favorable outcomes in defects with preserved bony walls and limited circumferential involvement [8,11,31,32,33,34]. In this case, however, the defect exhibited combined vertical and horizontal bone loss, buccal plate deficiency, and compromised implant positioning within the esthetic zone. These factors substantially diminished the probability of achieving stable hard- and soft-tissue regeneration through salvage procedures alone. Current decision-making frameworks indicate that when defect morphology and implant positioning are unfavorable, staged explantation and reconstruction provide greater long-term predictability than repeated regenerative attempts [13,35].

A key biological consideration in this case was the preservation of the maxillary canine. In situations where implants fail in areas with severe bone loss and missing adjacent teeth, extracting a strategically important tooth can further compromise interproximal bone architecture. The periodontal ligament of a natural tooth maintains vascular supply, proprioceptive function, and vertical bone height, all of which contribute to soft-tissue stability and papilla support [36,37]. Loss of this ligament-mediated bone homeostasis after extraction results in remodeling and flattening of the interproximal bone peak.

Although periodontal involvement occurred secondary to peri-implant inflammation, preservation of the canine was thought biologically advantageous. This consideration is especially significant in the anterior maxilla, where interproximal bone height is essential for papilla support and esthetic stability. Retaining the canine and performing guided tissue regeneration on its root surface maintained vertical bone architecture and papillary support throughout staged implant reconstruction. This strategy promoted stable soft-tissue contours and minimized the risk of papillary collapse and esthetic compromise during delayed implant placement.

Recent evidence demonstrates that both tooth preservation and implant placement can achieve high long-term survival rates. However, preserving natural dentition should be prioritized when feasible, as it prevents additional bone loss after extraction and maintains proprioceptive periodontal function [38,39]. Studies indicate that periodontal regeneration and comprehensive periodontal therapy improve the prognosis of periodontally compromised teeth, potentially delaying or avoiding the need for extraction and implant replacement, and highlight the importance of proper occlusal load [38,39,40]. Furthermore, retaining a natural canine reduces the number of required implants, simplifies prosthetic planning, and preserves occlusal guidance and esthetic harmony in the anterior maxilla [41,42].

Prosthetic planning, alongside surgical decision-making, was essential for selecting the appropriate treatment. In this case, the adjacent teeth had previously been restored with crowns; however, the patient expressed dissatisfaction with the esthetic result. Consequently, a comprehensive prosthetic re-evaluation was necessary. While a conventional fixed partial prosthesis was an option, this method would have imposed additional loading on teeth with reduced periodontal support. Conversely, implant-supported single crowns facilitated a biologically driven and prosthetically guided rehabilitation, enabling independent restoration of each unit, improved load distribution, and precise control of the emergence profile. This strategy was particularly important in the esthetic zone, where maintaining soft-tissue stability and papilla preservation is essential for long-term success.

Maintaining the interproximal bone peak is essential for esthetic stability. Both classical and contemporary studies demonstrate a direct relationship between bone crest height and papilla presence, as well as an increased risk of recession and black triangles when interproximal support is inadequate [43,44,45]. While bone grafting can restore ridge volume, recreating a true interproximal bone peak equivalent to that of a natural tooth remains biologically challenging and unpredictable. In this case, preservation of the canine provided a stable vertical reference and minimized the need for complex vertical regenerative procedures.

A natural tooth with an intact periodontal ligament preserves vertical interproximal bone height, which is crucial for soft-tissue support, papilla formation, and esthetic stability [45,46]. Although bone grafting procedures can restore ridge volume, regenerating a true interproximal bone peak comparable to that maintained by a natural tooth remains unpredictable [47,48,49].

Guided tissue regeneration (GTR) around the compromised adjacent teeth was incorporated into the staged protocol. Root surface conditioning with EDTA, application of enamel matrix derivative, and coverage with autogenous bone chips were employed to promote selective repopulation of periodontal ligament cells and true periodontal regeneration rather than repair. Current regenerative periodontal literature supports the long-term stability of teeth treated with these approaches, often demonstrating survival outcomes comparable to implant therapy when appropriate maintenance is provided [50,51]. The favorable regenerative outcome observed in this case was likely multifactorial. Successful GBR depends on maintaining space, maintaining clot stability, achieving primary wound closure, and ensuring the biological compatibility of graft materials. Composite grafting with autogenous bone (providing osteogenic and osteoinductive properties) combined with a volume-stable xenograft (providing scaffold stability) follows contemporary biologically driven regenerative principles [18,19,20]. Rigid membrane stabilization and tension-free closure were critical to preventing micromotion and soft-tissue invasion.

Minimally invasive explantation using reverse-torque techniques further contributed to preservation of residual defect walls and surrounding soft tissues, thereby maintaining regenerative potential [52]. Soft-tissue thickening with a connective tissue graft enhanced peri-implant mucosal stability and keratinized tissue width, factors associated with improved long-term peri-implant health [21,22,25]. Controlled provisionalization with milled PMMA restorations protected the regenerative site from functional loading while allowing soft-tissue conditioning, supporting the concept that prosthetic management is inseparable from surgical success [53,54,55].

In addition to surgical considerations, prosthetic design played an essential role in maintaining peri-implant soft-tissue stability. In this case, screw-retained zirconia restorations were fabricated on LMDT abutments, which are specifically designed to work with a convergent intramucosal neck, promoting increased soft-tissue thickness, improved esthetic integration, and protection of the underlying bone [56,57,58]. When used in conjunction with the biologically oriented preparation technique (BOPT), this design encourages soft-tissue adaptation and stabilization around the implant [59,60]. The hybrid-level implant design, featuring an anodized transmucosal neck, further contributed to stable soft-tissue adaptation and favorable peri-implant tissue integration.

Provisional restorations were digitally designed to establish a gradual and biologically guided emergence profile, transitioning from a concave subgingival contour to a slightly convex transmucosal profile. This approach supported soft-tissue maturation, promoted papilla formation, and minimized pressure on peri-implant tissues during healing [61]. The definitive restorations maintained these contours, ensuring long-term soft-tissue stability, improved cleansability, and favorable esthetic integration.

In summary, this case illustrates a biologically driven, staged approach for the management of advanced peri-implantitis when defect morphology and implant positioning limit regenerative predictability. Preservation of a compromised natural tooth in conjunction with guided tissue regeneration (GTR) may help maintain the vertical interproximal bone peak and papillary stability, thereby enhancing esthetic outcomes during delayed implant reconstruction. Radiographic evaluation at follow-up, although limited to the short term, demonstrated stable marginal bone levels around the implants, with no signs of progressive bone loss or peri-implant radiolucency.

Rather than focusing solely on implant salvage, integrating explantation, guided bone regeneration, guided tissue regeneration, soft-tissue augmentation, and prosthetically guided re-implantation may improve long-term stability and esthetic outcomes.

## 4. Limitations of the Study

This study has several limitations that should be acknowledged. First, it represents a single clinical case, limiting the generalizability of the findings. Although the clinical and radiographic outcomes were favorable, conclusions regarding the predictability and long-term success of the described treatment protocol cannot be extended to all cases of advanced peri-implantitis.

Second, the follow-up period after definitive prosthetic rehabilitation was relatively short, and long-term outcomes related to peri-implant tissue stability, marginal bone levels, and prosthetic complications could not be fully assessed. Calibrated marginal bone level measurements were not systematically recorded due to the clinical nature of this case and the use of different radiographic modalities. Extended follow-up is necessary to confirm the durability of the regenerated hard and soft tissues and the survival of the re-implanted fixtures.

Finally, as no control or comparison group was included, it was not possible to directly compare the outcomes of staged implant removal and re-implantation with alternative treatment strategies, such as regenerative peri-implantitis therapy without explantation. Well-designed prospective studies and controlled clinical trials are required to evaluate further the indications, benefits, and limitations of staged reconstruction protocols following advanced peri-implantitis.

## 5. Conclusions

Advanced peri-implantitis with extensive bone loss is a condition in which implant salvage procedures often demonstrate limited predictability. The present case illustrates that implant removal, followed by staged guided bone regeneration, delayed re-implantation, and meticulous soft-tissue and prosthetic management may represent a biologically sound and predictable treatment strategy in selected cases.

Explantation facilitated comprehensive site decontamination and prosthetically guided ridge reconstruction. The combination of hard- and soft-tissue augmentation supported stable peri-implant tissues and sufficient keratinized mucosa. Preservation of strategically important adjacent teeth, including a compromised canine, contributed to the maintenance of alveolar bone architecture and esthetic stability.

Considering the limitations inherent to a single case report, staged explantation and reconstruction may serve as a reliable alternative to repeated regenerative procedures in selected cases of advanced peri-implantitis.

## Figures and Tables

**Table 1:** Clinical timeline according to CARE guidelines.

Time Point	Event	Key Details
Baseline (August 2024)	Initial evaluation and diagnosis	Clinical examination and CBCT revealed advanced peri-implantitis affecting two maxillary implants (sites 2.2. and 2.4.), with extensive vertical and horizontal bone loss and involvement of adjacent teeth (2.1., 2.3., 2.5.).
Week 0	Treatment planning and informed consent	Decision for staged treatment including implant explantation, guided bone regeneration (GBR), guided tissue regeneration (GTR) delayed re-implantation, and full maxillary prosthetic rehabilitation. Written informed consent obtained.
Week 1	Stage One Surgery: Implant removal, GBR, GTR	Explantation of failing implants, debridement of peri-implant defects, root surface decontamination of adjacent teeth, GTR, and GBR using a 50:50 mixture of autogenous bone and collagenated porcine xenograft, covered with a resorbable membrane and primary closure.
Weeks 1–2	Early postoperative healing	Uneventful healing; no infection or membrane exposure; sutures removed after 12 days.
Months 0–6	Healing phase after GBR	Patient rehabilitated with a non–load-bearing tooth-supported provisional restoration; grafted site protected from mechanical loading.
Month 6	CBCT evaluation	CBCT demonstrated successful horizontal bone regeneration with an alveolar ridge width of approximately 8 mm and partial buccal bone coverage of adjacent canine root.
Month 6	Stage Two Surgery: Re-implantation and soft-tissue augmentation	Placement of two hybrid-level implants (sites 2.2. and 2.4.), additional lateral bone augmentation, and connective tissue grafting to increase keratinized mucosa, followed by tension-free primary closure.
Months 12	Stage Three Surgery: Implant uncovering	Implant exposure and placement of healing abutments; evaluation of peri-implant soft tissue stability.
Months 12–13	Provisional prosthetic phase	Fabrication and delivery of PMMA provisional restorations; soft tissue conditioning and maturation monitored.
Months 13	Definitive prosthetic rehabilitation	Delivery of individual zirconia crowns for full maxillary rehabilitation, including implant-supported and tooth-supported restorations.
Short-term follow-up (3 weeks after provisional delivery)	Soft tissue evaluation	Satisfactory peri-implant soft tissue maturation and adequate width of keratinized gingiva observed.
Follow-up (after definitive restoration)	Outcome assessment	Clinical and radiographic evaluation confirmed stable implants, healthy peri-implant tissues, proper prosthetic adaptation, and favorable esthetic and functional outcomes.
6 months after final prosthetic rehabilitation	Final follow-up and outcome assessment	Clinical and radiographic evaluation confirmed stable peri-implant and periodontal conditions, probing depths ≤ 3 mm at implant and adjacent tooth sites, absence of bleeding on probing or suppuration, maintenance of marginal bone levels on periapical and bitewing radiographs, and stable prosthetic function and esthetics.

## Data Availability

The data presented in this study are available on request from the corresponding authors. The data are not publicly available due to ethical restrictions and patient privacy considerations.

## Abbreviations

The following abbreviations are used in this manuscript:

BOP	Bleeding on Probing
BOPT	Biologically Oriented Preparation Technique
CBCT	Cone-Beam Computed Tomography
GBR	Guided Bone Regeneration
GTR	Guided Tissue Regeneration
LMDT	Low-MDT—Management of Deep Tissue
OPG	Orthopantomogram
PI	Plaque Index
PGCL	Polyglycolide-Caprolactone
PMMA	Polymethyl Methacrylate
PPD	Periodontal Probing Depth
PTFE	Polytetrafluoroethylene
